# Impaired Translocation of GLUT4 Results in Insulin Resistance of Atrophic Soleus Muscle

**DOI:** 10.1155/2015/291987

**Published:** 2015-02-03

**Authors:** Peng-Tao Xu, Zhen Song, Wen-Cheng Zhang, Bo Jiao, Zhi-Bin Yu

**Affiliations:** Department of Aerospace Physiology, Fourth Military Medical University, No. 169 Changlexi Road, Xi'an 710032, China

## Abstract

Whether or not the atrophic skeletal muscle induces insulin resistance and its mechanisms are not resolved now. The antigravity soleus muscle showed a progressive atrophy in 1-week, 2-week, and 4-week tail-suspended rats. Hyperinsulinemic-euglycemic clamp showed that the steady-state glucose infusion rate was lower in 4-week tail-suspended rats than that in the control rats. The glucose uptake rates under insulin- or contraction-stimulation were significantly decreased in 4-week unloaded soleus muscle. The key protein expressions of IRS-1, PI3K, and Akt on the insulin-dependent pathway and of AMPK, ERK, and p38 on the insulin-independent pathway were unchanged in unloaded soleus muscle. The unchanged phosphorylation of Akt and p38 suggested that the activity of two signal pathways was not altered in unloaded soleus muscle. The AS160 and GLUT4 expression on the common downstream pathway also was not changed in unloaded soleus muscle. But the GLUT4 translocation to sarcolemma was inhibited during insulin stimulation in unloaded soleus muscle. The above results suggest that hindlimb unloading in tail-suspended rat induces atrophy in antigravity soleus muscle. The impaired GLUT4 translocation to sarcolemma under insulin stimulation may mediate insulin resistance in unloaded soleus muscle and further affect the insulin sensitivity of whole body in tail-suspended rats.

## 1. Introduction

The skeletal muscle has high plasticity to adapt to the environmental alterations. Weightlessness or simulated weightlessness, denervation, tenotomy, long-term of immobilization, and bed rest induce a disused atrophy in skeletal muscles [1–6]. Soleus muscle denervation induces the tissue-specific insulin resistance in which the mechanism is involved in the decreased expression and phosphorylation in the pivotal downstream signaling molecules of insulin receptor including insulin receptor substrate-1 (IRS-1), phosphatidylinositol 3-kinase (PI3K), and protein kinase B (Akt) [1, 7]. Seven days of immobilization reduce insulin-stimulated phosphorylation of IR-*β*, IRS-1 and PI3K which induce insulin resistance of the skeletal muscle [2]. Tenotomy, bed rest, and space flight also induce insulin resistance in antigravity skeletal muscles [4–6]. There are the controversy reports on the relationship between insulin resistance and the unloaded soleus muscle of tail-suspension. O'Keefe and colleagues reported that whole-body glucose tolerance and insulin action on soleus muscle glucose transport was decreased in 1 day of tail-suspended rats. The development of insulin resistance in the 1-day unloaded soleus is not due to impaired functionality of elements involved in the IR/IRS-1/PI3-kinase/Akt signaling pathway. However, activation of the stress-activated p38 mitogen-activated protein kinase (p38 MAPK) may play a role in this response [8]. In contrast, O'Keefe and colleagues also reported that the increased insulin action on glucose transport in the 7-day unloaded soleus is associated with increased insulin signaling through IRS-1 and PI3K and decreased p38 MAPK protein expression [9]. Hilder and colleagues found that elevated activity of c-Jun NH_2_-terminal kinase (JNK) phosphorylated IRS-1 at Ser307 and further reduced Akt activity, providing biochemical evidence for the development of insulin resistance in 2-week to 13-week unloaded soleus muscle [10]. Adenosine 5′-monophosphate-activated protein kinase (AMPK) is also an important mediator of insulin-independent glucose uptake in atrophic soleus muscle [11]. Thus, denervation, tenotomy, immobilization, and long-term bed rest induce insulin resistance in atrophic skeletal muscles. But whether or not the tail-suspension results in insulin resistance in unloaded soleus muscle and its mechanisms is not resolved now. Insulin resistance may be involved in insulin-dependent and insulin-independent signal transduction pathways in unloaded soleus muscle.

Therefore, the aims of this study were to observe whole-body glucose tolerance and insulin action on glucose transport of unloaded soleus muscle in the rat. The expression and activity of pivotal proteins on insulin-dependent and insulin-independent signal transduction pathways were measured and the mechanisms of insulin resistance were elucidated in unloaded soleus muscle.

## 2. Methods

### 2.1. Tail-Suspended Rat Model

Healthy male Sprague-Dawley rats weighing 220 ± 10 g were randomly divided into the synchronous control (CON) and tail-suspended (SUS). Tail-suspension was performed using a Morey-Holton method for 1 week, 2 weeks, and 4 weeks, respectively [12]. Care was taken to protect the tail tissue, and the movement of the rats was not restricted during the procedure. There were 6 rats in each group. All rats were housed in a 20 ± 2°C environment with a 12:12 h light-dark cycle and were fed with rat chow and water* ad libitum*. All animal procedures were approved by the Animal Care and Use Committee at the Fourth Military Medical University.

### 2.2. Hyperinsulinemic-Euglycemic Clamps

Insulin clamps were performed as previously described [13]. Rats were fasted overnight. Rats were anesthetized, and catheters were inserted in left jugular vein and right carotid artery. The externalized rat catheters were connected to catheter extensions attached to infusion syringes. Just prior to the onset of the insulin clamp, an arterial blood sample (5 *μ*L) was obtained to evaluate levels of arterial glucose (OMRON HEA-214, Abbott Diabetes Care Inc., USA). A primed continuous intravenous infusion of human insulin (8 mU/kg/min; Sigma-Aldrich Co., USA) was continued for 120 minutes. Glucose solution (20%) was infused by intravenous at a variable rate to maintain blood glucose between 5 and 6 mM. Arterial blood samples were taken at 5-minute intervals to monitor plasma glucose concentrations. While euglycemia was achieved for at least 30 minutes, the steady-state blood glucose and glucose infusion rate were measured for 60 min at 10-minute intervals. Erythrocytes were suspended in saline and reinjected into the rats to prevent a fall in hematocrit and to minimize stress.

### 2.3. Glucose Uptake in Skeletal Muscle [14]

^18^F-2-fluoro-D-deoxyglucose (^18^F-FDG) was synthetized by a CYCLONE 18/9 cyclotron (IBA, Belgium). Rats were fasted overnight but with free access to water. Rats were divided into the control group, insulin stimulation group, and contraction stimulation group. There are three rats in each group. Rats received intraperitoneal injections of insulin (2 mU/g body weight) as the insulin stimulation group. A dose of ^18^F-FDG (74 MBq) was injected in the tail vein after insulin injection under sodium pentobarbital (40 mg/kg body weight) anesthesia. The baseline ^18^F-FDG uptake in the control group was assessed without insulin injection. The left sciatic nerve was carefully exposed and isolated at the midthigh level. A pair of silver hook electrodes was placed under the sciatic nerve for electrical stimulation, and the exposed nerve was covered with warm mineral oil. The left hindlimb was fixed at a natural position to induce tetanic contraction of skeletal muscles during the sciatic nerve stimulation. After injection of ^18^F-FDG without insulin treatment, sciatic nerve was stimulated at 100 Hz and suprathreshold intensity (300 *μ*A) with rectangular pulses of 0.5 ms duration at 5-minute intervals. Tetanic contraction was produced under intermittent stimulation of 1 s intervals at a 50% duty cycle (one 500 ms contraction every 1,000 ms) for 5 min [15]. The stimulated left hindlimb was as the contraction stimulation group and non-stimulated right hindlimb as the control group.

Soleus and extensor digitorum longus (EDL) muscles were collected at the 60th minute after ^18^F-FDG injection. After weighing the muscle samples, the radioactivity was measured using a CRC-15PET Dose Calibrator (Capintec, USA). Skeletal muscle ^18^F-FDG uptake was then expressed as the standard uptake value (SUV) obtained by calculating the ratio of muscle ^18^F-FDG activity to injected dose normalized to the body weight as the following equation:

(1) SUV=Radioactivity  concentration  in  the  muslce MBq/gInjected  dose MBq/weight  of  rat (g).

### 2.4. Plasma Membrane Protein Extraction of Skeletal Muscle

Plasma membrane protein of soleus muscle was extracted using a plasma membrane protein extraction kit (Catalog number K268-50, BioVision Inc., USA) and following the manufacturer's protocols. Briefly, the muscle tissue (100 mg) was homogenized on ice by a Polytron (PT-MR 2100, CE) in Homogenize Buffer with 1 mM PMSF. The homogenates were centrifuged at 700 ×g for 10 min and then the supernatant was centrifuged at 14,000 ×g for 30 min at 4°C. The total membrane proteins pellet was resuspended in the Upper Phase Solution with 1 mM PMSF, then added the Lower Phase Solution, incubated on ice for 5 min, and centrifuged at 1000 ×g for 5 min at 4°C. The upper phase was carefully collected, then centrifuged at 14,000 ×g for 10 min at 4°C. The resulting pellet which contained the plasma membrane protein (PM) was stored for subsequent Western blotting.

### 2.5. Western Blotting Analysis

As described previously [16], total protein was extracted from rat soleus muscle by homogenization in SDS-PAGE sample buffer containing 1% SDS. The muscle protein extracts were resolved by SDS-PAGE using Laemmli gels. A 10% gel with an acrylamide/bisacrylamide ratio of 37.5 : 1 was used for the examination of IRS-1, PI3K, and AS160; and a 12% gel with an acrylamide/bisacrylamide ratio of 29 : 1 was used for the examination of Akt, p38, ERK, GLUT4 and *β*-actin. After electrophoresis, proteins were electrically transferred to nitrocellulose membrane (0.45 *μ*m pore size) using a Bio-Rad semidry transfer apparatus. The blotted nitrocellulose membranes were blocked with 1% bovine serum albumin (BSA) in Tris-buffered saline (TBS; 150 mM NaCl, 50 mM Tris-HCl, pH 7.5) and incubated with anti-IRS-1, anti-PI3K, anti-Akt, anti-p-Akt (phosphorylation at Ser473), anti-AMPK, anti-p38, anti-p-p38 (phosphorylation at Thr180 and Tyr182), anti-ERK (1 : 500 dilution; Santa Cruz Biotechnology, Inc., CA, USA); anti-AS160, anti-GLUT4 (1 : 2000 dilution; Cell Signaling Technology, Inc., Danvers, MA, USA) or mouse monoclonal anti-*β*-actin (1 : 2000 dilution; Sigma-Aldrich) in TBS containing 0.1% BSA at 4°C overnight. The nitrocellulose membranes were incubated with IRDye 680CW goat-anti mouse or IRDye 800CW goat-anti-rabbit secondary antibodies (1 : 10,000 dilution) for 90 min at room temperature, and visualized using an Odyssey scanner (LI-COR Biosciences, Lincoln, NE, USA). Quantification analysis of blots was performed with the NIH Image J software.

### 2.6. Immunofluorescent Cytochemistry and Confocal Analysis

Soleus muscle samples from the rats with or without insulin stimulation were cut from the middle of the muscle belly and frozen with optimum cutting temperature compound and isopentane in liquid nitrogen. Frozen 10 *μ*m-thick muscle cross sections were obtained in a freezing cryostat at −20°C. The sections were air dried at room temperature, fixed in ice-cold acetone for 30 min, permeabilized in the phosphate buffered saline (PBS; 135 mM NaCl, 10 mM sodium phosphate pH 7.0) including 0.1% Triton X-100 for 30 min, and blocked in 1% bovine serum albumin (BSA) in PBS for 60 min at room temperature. Sections were incubated with anti-GLUT4 (1 : 100 dilution) at 4°C overnight. The slides were rinsed twice in PBS and incubated with rhodamine-labeled goat-anti-rabbit IgG (1 : 400 dilution; Invitrogen, Carlsbad, USA) for 60 min. Stained sections were observed using a laser-scanning confocal microscope equipped with the FV10-ASW system (Olympus FV1000). The rhodamine-labeled signals were visualized at 519 nm. Images were acquired at a 60x water objective. Optical densitometry analysis of GLUT4 was performed using Olympus Fluoview image analysis software (Olympus Co., Ltd.). Nuclei were identified by the Hoechst 33258 (1 : 100 dilution; Invitrogen) staining.

### 2.7. Statistical Analysis

Data are presented as mean ± SEM. Differences between the synchronous control and tail-suspended groups were compared by the paired Student's *t*-test. For multi-group comparisons, one-way ANOVA followed by Dunnett posthoc test was performed. Probability values of *P* < 0.05 were considered statistically significant.

## 3. Results

### 3.1. Atrophy in Soleus Muscles, but Not in EDL Muscles after Hindlimb Unloading

There was no difference in body weight between 1-week and 2-week tail-suspended (SUS) groups and their synchronous control (CON) groups (Table 1). Body weight in 4-week SUS group showed a significant decrease (*P* < 0.05). The ratios of soleus weight to body weight showed a progressive decrease in 1-week, 2-week, and 4-week SUS groups (*P* < 0.05 or *P* < 0.01), but not change in the ratios of EDL weight to body weight (Table 1).

### 3.2. Insulin Sensitivity of Whole Body and Glucose Uptake Rate in Soleus and EDL Muscles

**Figure 1:**
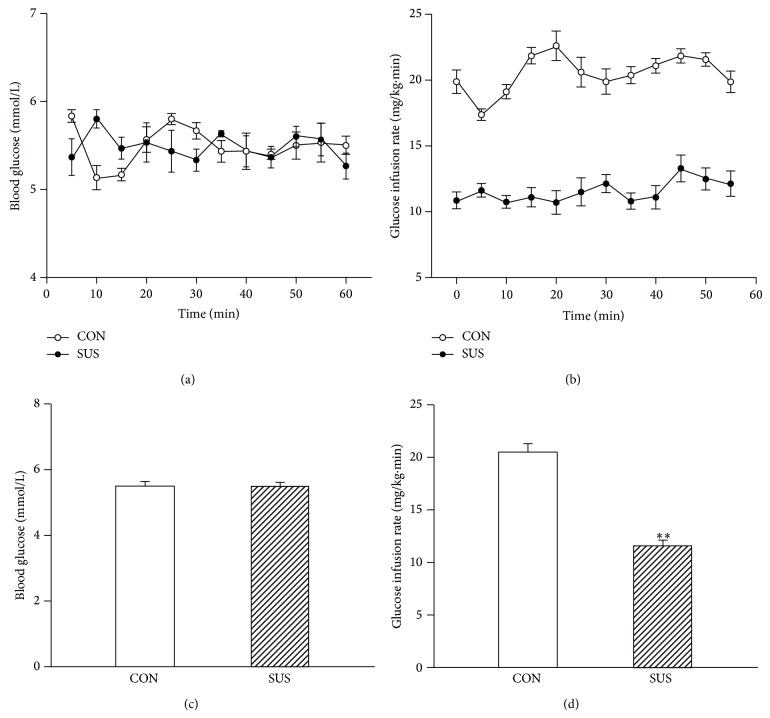
The insulin sensitivity of whole body during a 60-min hyperinsulinemic-euglycemic clamp experiment in the control (CON) and 4-week tail-suspended (SUS) rats. (a) Blood glucose. (b) Glucose infusion rate. (c) Average blood glucose level during 60-min clamp. (d) Average glucose infusion rate during 60-min clamp. Values are mean ± SEM, *n* = 6 rats for each group. ^**^ *P* < 0.01* versus* CON group.

Following overnight fast, arterial blood glucose was comparable between the control and 4-week tail-suspended groups. To examine insulin action in the control and 4-week tail-suspended rats, insulin clamps were performed. During the last 60 min of the insulin clamp, arterial blood glucose levels were held constant in CON and SUS groups (Figures 1(a) and 1(c)). Analysis of glucose infusion rates (GIR) showed considerable insulin resistance in SUS group, with rats disposing of about 40% less glucose compared with CON (*P* < 0.01) (Figures 1(b) and 1(d)).

**Figure 2:**
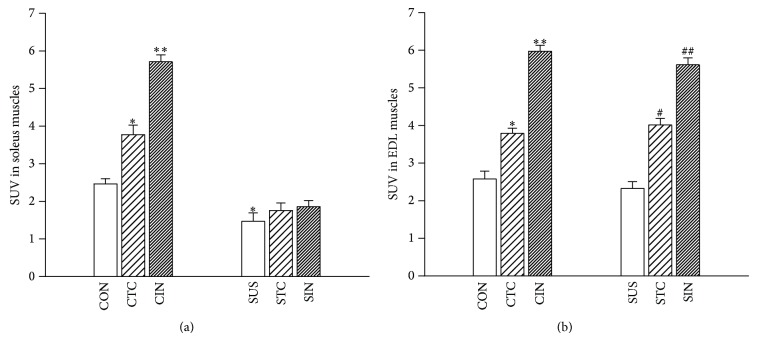
Effects of tetanic contraction and insulin stimulation on the glucose uptake in the control and unloaded soleus and EDL muscles. (a) Glucose uptake of soleus muscles. (b) Glucose uptake in EDL muscles. CTC, tetanic contraction of CON muscle. CIN, CON muscle treated by insulin. STC, tetanic contraction of SUS muscle. SIN, SUS muscle treated by insulin. Values are mean ± SEM, *n* = 6 samples for each group. ^*^ *P* < 0.05 or ^**^ *P* < 0.01* versus* CON group. ^#^ *P* < 0.05 or ^##^ *P* < 0.01* versus* SUS group.

Tissue-specific glucose utilization showed lower rates of glucose uptake in unloaded soleus muscles compared with the CON group under basic condition (*P* < 0.05) (Figure 2(a)). Tetanic contraction of soleus muscle induced a significant increase in glucose uptake (*P* < 0.05) and insulin stimulation induced a further increase in glucose uptake (*P* < 0.01), but not in unloaded soleus muscles after 4-week tail-suspension (Figure 2(a)). However, there were no significant differences in glucose uptake of EDL muscles with contraction stimulation and with or without insulin stimulation between CON and SUS groups (Figure 2(b)).

### 3.3. Protein Expression of Elements on Insulin-Dependent or Insulin-Independent Signal Pathway in Soleus Muscles

**Figure 3:**
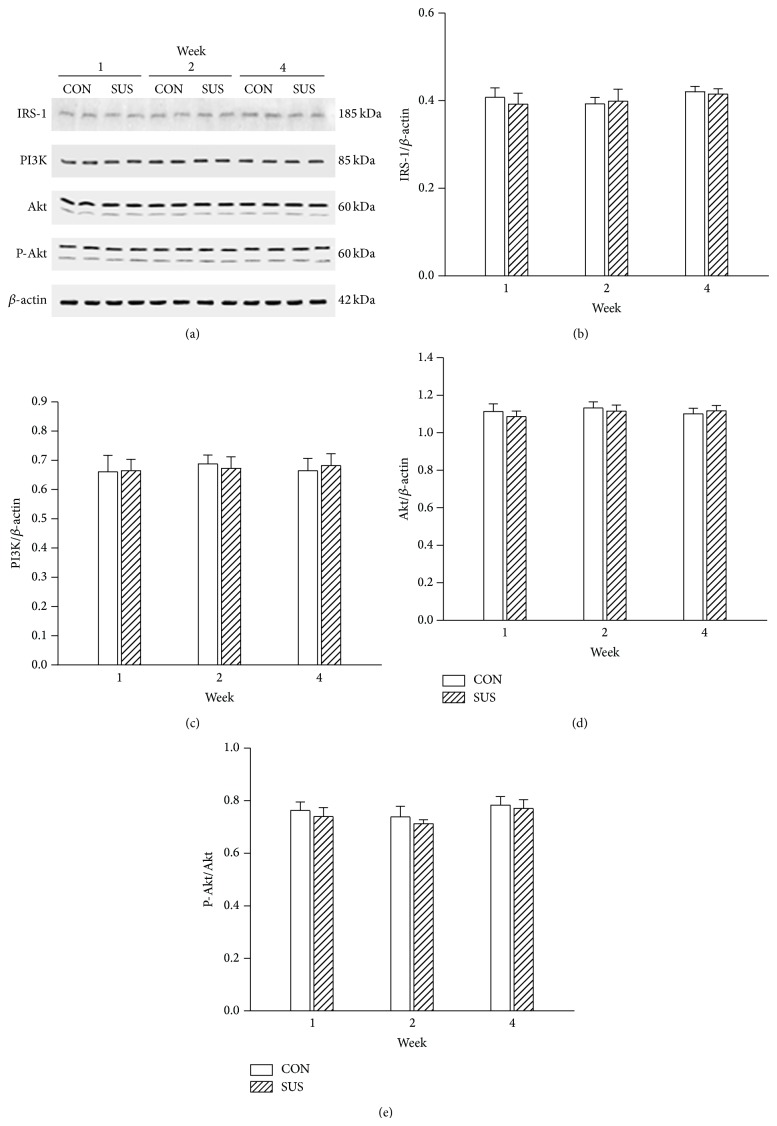
Expression of IRS-1, PI3K, Akt, and P-Akt in the unloaded soleus of rats. (a) Representative Western blots. (b) Ratios of IRS-1 to *β*-actin. (c) Ratios of PI3K to *β*-actin. (d) Ratios of Akt to *β*-actin. (e) Ratios of P-Akt to total Akt. Values are mean ± SEM, *n* = 6 samples for each group.

IRS-1, PI3K, and Akt are key elements on insulin-dependent signal transduction pathway. The protein expression of IRS-1, PI3K, and Akt was not changed in 1-week, 2-week, and 4-week unloaded soleus muscles compared with the synchronous control groups (Figure 3). Akt is a pivotal molecule regulated the downstream AS160. The unchanged phosphorylation of Akt indicated that insulin-dependent signal pathway activity was not altered in unloaded soleus muscles (Figure 3(e)).

**Figure 4:**
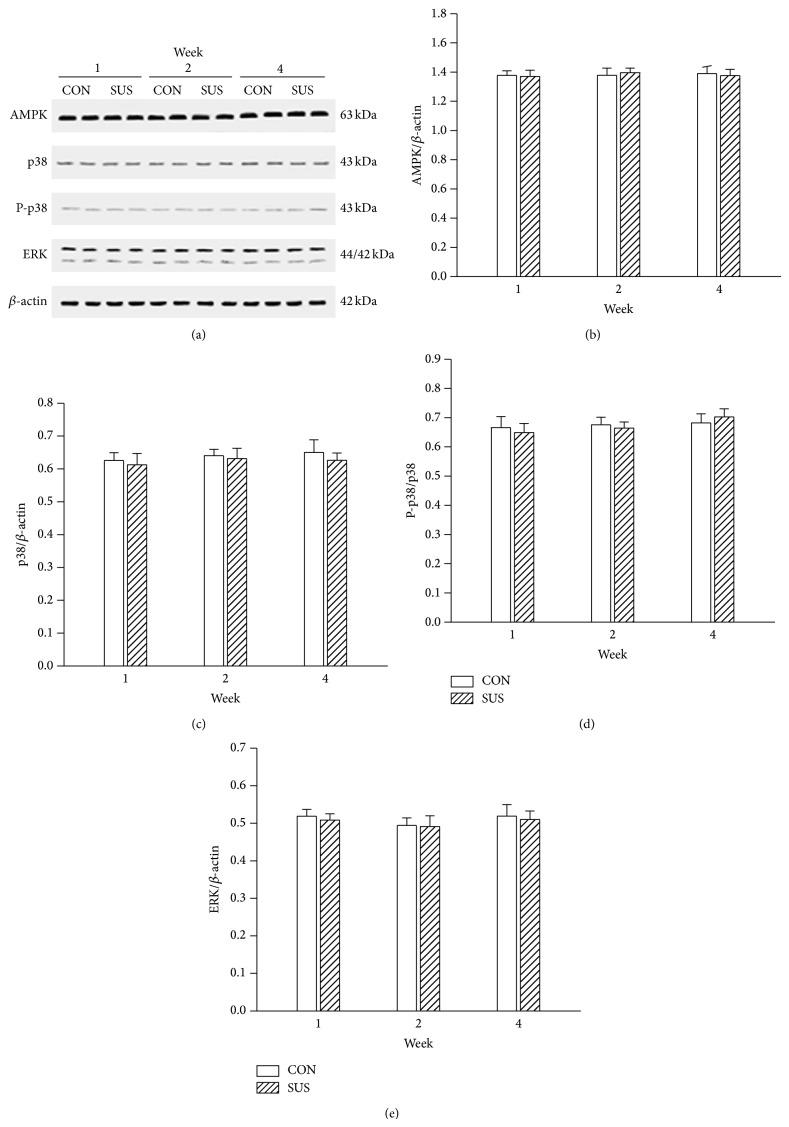
Expression of AMPK, ERK, p38, and P-p38 in the unloaded soleus of rats. (a) Representative Western blots. (b) Ratios of AMPK to *β*-actin. (c) Ratios of p38 to *β*-actin. (d) Ratios of P-p38 to p38. (e) Ratios of ERK1/2 to *β*-actin. Values are mean ± SEM, *n* = 6 samples for each group.

AMPK, p38, and ERK may be key elements on insulin-independent signal transduction pathway. The expression of AMPK, p38, and ERK was also unchanged in 1-week, 2-week, and 4-week unloaded soleus muscles compared with the synchronous control groups (Figure 4). The p38 can modulate the downstream AS160. The comparable phosphorylation level of p38 between the CON and SUS groups indicated that insulin-independent signal pathway activity was unchanged in unloaded soleus muscles (Figure 4(d)).

### 3.4. Protein Expression of Elements on Common Downstream Signal Pathway in Soleus Muscles

**Figure 5:**
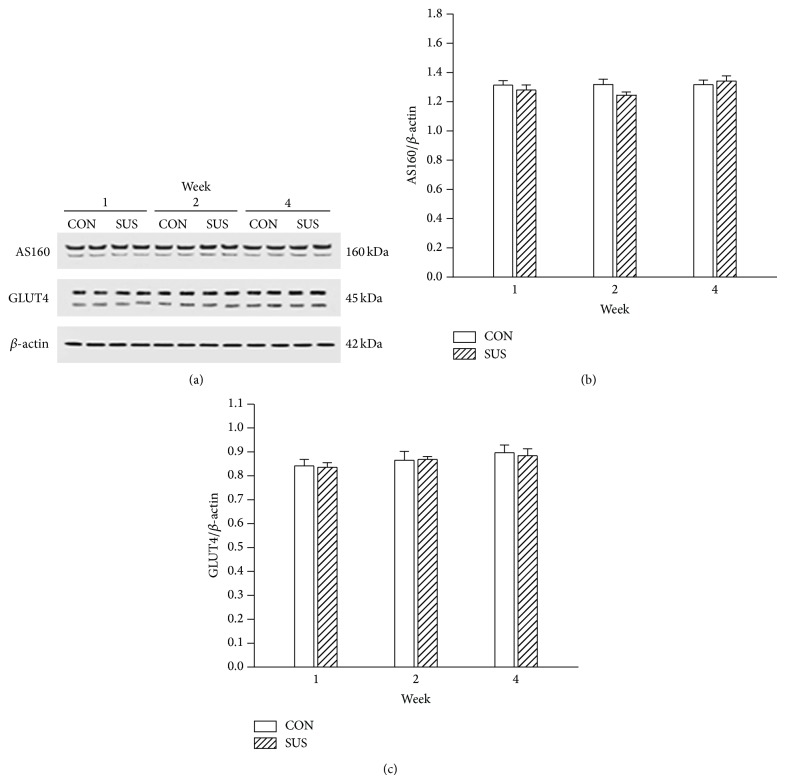
Expression of AS160 and GLUT4 in the unloaded soleus of rats. (a) Representative Western blots. (b) Ratios of AS160 to *β*-actin. (c) Ratios of GLUT4 to *β*-actin. Values are mean ± SEM, *n* = 6 samples for each group.

The insulin-dependent and insulin-independent signal pathways converge on a common downstream signal pathway which mainly consists of AS160 and GLUT4. The expression of AS160 and GLUT4 was not altered in 1-week, 2-week, and 4-week unloaded soleus muscles compared with the synchronous control groups (Figure 5).

### 3.5. GLUT4 Translocation to Sarcolemma in Soleus Muscles

**Figure 6:**
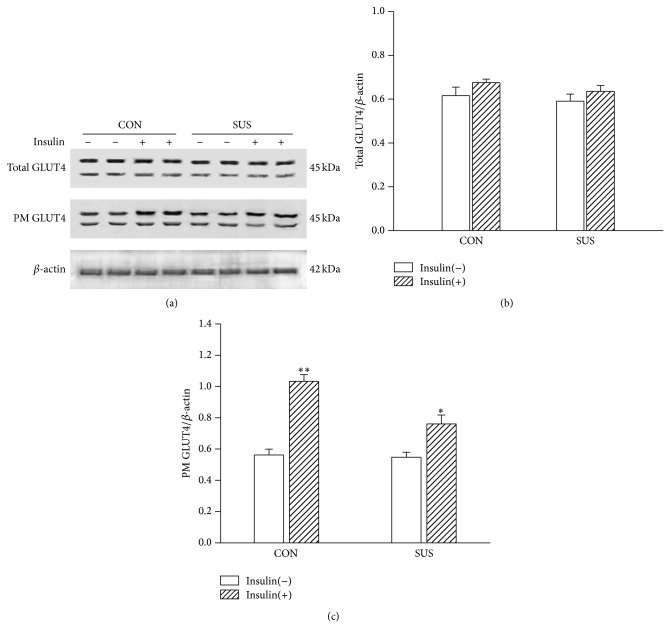
Changes in GLUT4 of plasma membrane fraction (PM) under insulin stimulation in the unloaded soleus of rats. (a) Representative Western blots. (b) Ratios of total GLUT4 to *β*-actin. (c) Ratios of PM GLUT4 to *β*-actin. ^*^ *P* < 0.05* versus* control with insulin stimulation group [insulin(+)], ^**^ *P* < 0.01* versus* control without insulin stimulation group [insulin(−)]. Values are mean ± SEM, *n* = 6 samples for each group.

BioVision's kit was designed to not only extract the total cellular membrane proteins, but also purify specifically the plasma membrane proteins over 90%. The total GLUT4 was detected using the original homogenates of soleus muscles. The expression of total GLUT4 of soleus muscle was not altered without or with insulin stimulation (Figure 6). The GLUT4 on the plasma membrane of soleus muscle fibers was increased after insulin stimulation in the control group (*P* < 0.01); it indicated increased GLUT4 translocation to the plasma membrane under insulin stimulation. However, the increment of GLUT4 translocation to the plasma membrane was less in unloaded soleus muscle than that in the control soleus muscle (Figure 6).

**Figure 7:**
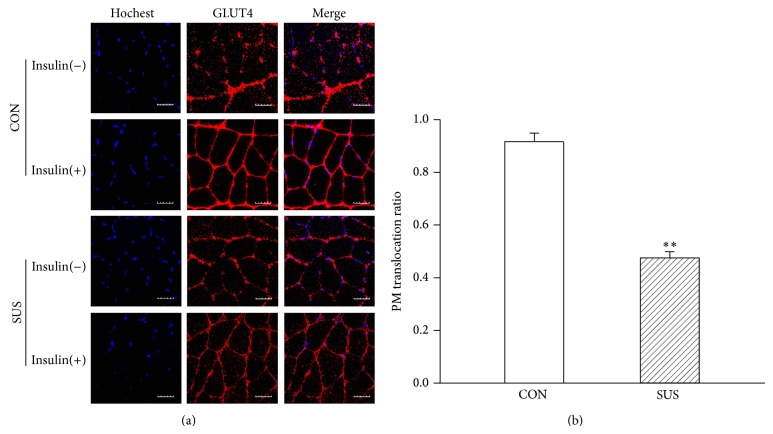
Changes in GLUT4 membrane translocation ratio under insulin stimulation in the unloaded soleus of rats. (a) Representative immunofluorescent sections. GLUT4 was stained in red fluorescent rhodamine. Nuclei were stained in blue by Hochest 33258. (b) Quantitative GLUT4 membrane translocation ratio. Insulin(+), with insulin stimulation. Insulin(−), without insulin stimulation. Scale bar, 5 *μ*m. Values are mean ± SEM, *n* = 30 muscle fibers for each group. ^**^ *P* < 0.01* versus* CON group.

To confirm GLUT4 translocation to the sarcolemma, immunofluorescent images of soleus muscles were observed. The cross section of soleus muscle fibers appeared in distinct GLUT4 puncta localized the sarcolemma and cytosol in the CON and SUS group without insulin stimulation. GLUT4 translocation induced by insulin resulted in an increased GLUT4-red fluorescent labeling of the sarcolemma and decreased GLUT4 vesicles of cytosol in the control soleus muscle fibers (Figure 7(a)). The ratios of GLUT4 translocation to the sarcolemma was less in unloaded soleus muscle than that in the control soleus muscle with insulin stimulation (Figure 7(b)).

## 4. Discussion

Insulin resistance is a physiological condition in which cells fail to respond to the normal actions of the hormone insulin. Insulin resistance also is a major risk factor for developing type 2 diabetes caused by the inability of insulin-target tissues to respond properly to insulin. Skeletal muscle occupied 40%~60% body weight is the major tissue for insulin-mediated glucose clearance. Therefore, insulin resistance in skeletal muscle is an important etiology of type 2 diabetes. Astronauts show a progressive atrophy in antigravity skeletal muscle. The atrophic skeletal muscle is related to insulin resistance. In order to maintain astronaut health the mechanisms underlying insulin resistance in skeletal muscle must be elucidated.

There are two signal transduction pathways for glucose transport in skeletal muscle. Insulin action involves a series of signaling cascades initiated by insulin binding to its receptor, eliciting receptor autophosphorylation and activation of the receptor tyrosine kinase, resulting in tyrosine phosphorylation of insulin receptor substrates (IRSs). Phosphorylation of IRSs lead to activation of phosphatidylinositol 3-kinase (PI3K) and, subsequently, to activation of Akt and its downstream mediator AS160, all of which are important steps for stimulating glucose transport induced by insulin [17]. Thus, the key elements in insulin-dependent signal transduction pathway include IRS, PI3K, Akt, AS160 and GLUT4. Contraction of skeletal muscle also stimulates glucose transport mediated by insulin-independent signal transduction pathway [18]. Contraction of skeletal muscle activates 5′-monophosphate-activated protein kinase (AMPK) by consumption of intracellular ATP [19]. AMPK can activate the downstream multiple kinases including p38, ERK1/2, or AS160 [20]. Thus, the key elements on insulin-independent pathway may include AMPK, p38, AS160 and GLUT4. Obviously, the distinct signaling pathways that are stimulated by insulin and exercise/contraction converge at AS160 [21].

In the present study protein expression of IRS-1, PI3K and Akt on insulin-dependent signal pathway and of AMPK, ERK, and p38 on insulin-independent signal pathway was not altered in unloaded soleus muscles. The expression of AS160 and GLUT4 on the common downstream pathway also remained unchanged. Phosphorylation of Akt and p38 unchanged. But the insulin-stimulated membrane translocation of GLUT4 was inhibited in unloaded soleus muscle. These results suggested that both insulin-dependent and insulin-independent signal pathways were not impaired in unloaded soleus muscle. The inhibition of GLUT4 translocation might be a major cause of insulin resistance in unloaded soleus muscle. However, the mechanisms underlying the inhibition of GLUT4 translocation were not completely understood in unloaded skeletal muscle.

The magnitude of a dense membrane compartment of GLUT4 is correlated to the degree of the insulin-stimulated glucose uptake in skeletal muscles [22]. The magnitude of GLUT4 membrane translocation was influenced by the protein level of GLUT4. A lot of factors inhibit GLUT4 expression at the basic or insulin-stimulated conditions. Insulin infusion can increase GLUT4 expression in the health persons, obese one with insulin resistance and type 1 diabetic patients, but not in type 2 diabetic patients. GLUT4 expression was not stimulated by 3 hours of insulin stimulation in the subjects born with intrauterine growth retardation [22]. Moreover, the magnitude of the defect in GLUT4 mRNA regulation by insulin was correlated to the degree of insulin resistance [23]. Slow-twitch muscle fibers of vastus lateralis decreased in obese people compared with similar body weight of healthy people, and further declined in type 2 diabetic patients. Because GLUT4 density was significantly higher in slow-twitch than that of fast-twitch fibers in biopsy specimens from lean and obese subjects, a reduction in the fraction of slow-twitch fibers reduced the insulin-sensitive GLUT4 pool in type 2 diabetes and thus contributed to skeletal muscle insulin resistance [24]. Targeted disruption of GLUT4 selectively in muscle induced severe insulin resistance and glucose intolerance [25]. In contrast, overexpression of GLUT4 in skeletal muscle showed lowered blood glucose [26] and increased insulin- or contraction-stimulated glucose transport that could be of benefit in the treatment of type 2 diabetes [27].

Besides glucose uptake modulated by the amount of GLUT4, there are numerous reports that indicate that the activity of GLUT4 is regulated independently of its recruitment to the membrane [28]. Garvey et al. reported that regardless of whether subjects had diabetes, as long as there was insulin resistance, the translocation of intracellular GLUT4 to sarcolemma had been impaired, and the expression of GLUT4 was not significantly reduced [22]. Enhanced GLUT4 translocation increased obviously insulin sensitivity in skeletal muscles [29].

The above researches indicate that glucose uptake mediated by GLUT4 is not only the rate-limiting step for skeletal muscle glucose utilization in certain physiological states, but also the maintenance of whole body glucose homeostasis [25, 30]. Insulin resistance in skeletal muscle is due to the attenuated expression level of GLUT4 with or without insulin-stimulation, decreased GLUT4 activity or impaired GLUT4 translocation to sarcolemma. However, the GLUT4 translocation to sarcolemma may be a major obstacle. In this study GLUT4 expression levels was not changed in atrophic soleus muscle compared with the synchronous control, however, the impaired GLUT4 translocation to sarcolemma might be a major cause to induce insulin resistance in atrophic soleus muscle. The mechanisms of the inhibited GLUT4 translocation still need to be elucidated in future experiments.

Insulin- and contraction-stimulated glucose uptake was decreased in atrophic soleus muscle, but not changed in fast-twitch muscle EDL. The tail-suspension of rat could affect multiple antigravity slow-twitch muscles of hindlimb; therefore, whole body glucose uptake of rat was also reduced.

In conclusion, hindlimb unloading in tail-suspended rat resulted in atrophy in slow-twitch soleus muscle. Insulin- and contraction-stimulated glucose uptake decreased in atrophic soleus muscle and whole body glucose uptake was also affected in tail-suspended rats. The mechanisms of insulin resistance may be involved in the impaired GLUT4 translocation to sarcolemma in atrophic soleus muscle.

## Figures and Tables

**Table 1:** Changes in body weight, soleus weight, and EDL weight of control and tail-suspended rats.

Groups	CON1	CON2	CON4	SUS1	SUS2	SUS4
Body weight (g)	253.0 ± 2.3	281.0 ± 5.1	367.0 ± 2.6	255.0 ± 3.3	270.0 ± 2.8	356.0 ± 2.5^*^
SW (mg)	96.11 ± 1.2	112.2 ± 2.4	150.4 ± 2.2	76.5 ± 2.5^*^	61.1 ± 1.4^**^	53 ± 1.3^**^
SW/BW (%)	38.4 ± 0.6	40.8 ± 0.8	41.2 ± 0.3	30.8 ± 0.4^*^	23.1 ± 0.4^**^	15.5 ± 0.4^**^
EW/BW (%)	44.3 ± 0.3	43.2 ± 0.5	41.5 ± 0.7	45.2 ± 0.6	42.8 ± 0.3	40.1 ± 0.8

BW: body weight. SW: soleus weight. EW: EDL weight. Values are mean ± SEM, *n* = 6 rats in each group. ^*^ *P* < 0.05 or ^**^ *P* < 0.01 versus synchronous CON group.
